# A nutrition and conditioning intervention for natural bodybuilding contest preparation: case study

**DOI:** 10.1186/s12970-015-0083-x

**Published:** 2015-05-01

**Authors:** Scott Lloyd Robinson, Anneliese Lambeth-Mansell, Gavin Gillibrand, Abbie Smith-Ryan, Laurent Bannock

**Affiliations:** Guru Performance LTD, 58 South Molton St, London, W1K 5SL UK; Institute of Sport & Exercise Science, University of Worcester, Henwick Grove, Worcester, WR2 6AJ UK; Ultimate City Fitness, 1-3 Cobb Street, London, E1 7LB UK; Department of Exercise and Sport Science, University of North Carolina Chapel Hill, Office: 303A Woolen, 209 Fetzer Hall, Chapel Hill, NC USA

**Keywords:** Sports nutrition, Physique, Conditioning, Body composition, Fat oxidation, Metabolic health

## Abstract

Bodybuilding competitions are becoming increasingly popular. Competitors are judged on their aesthetic appearance and usually exhibit a high level of muscularity and symmetry and low levels of body fat. Commonly used techniques to improve physique during the preparation phase before competitions include dehydration, periods of prolonged fasting, severe caloric restriction, excessive cardiovascular exercise and inappropriate use of diuretics and anabolic steroids. In contrast, this case study documents a structured nutrition and conditioning intervention followed by a 21 year-old amateur bodybuilding competitor to improve body composition, resting and exercise fat oxidation, and muscular strength that does not involve use of any of the above mentioned methods. Over a 14-week period, the Athlete was provided with a scientifically designed nutrition and conditioning plan that encouraged him to (i) consume a variety of foods; (ii) not neglect any macronutrient groups; (iii) exercise regularly but not excessively and; (iv) incorporate rest days into his conditioning regime. This strategy resulted in a body mass loss of 11.7 kg’s, corresponding to a 6.7 kg reduction in fat mass and a 5.0 kg reduction in fat-free mass. Resting metabolic rate decreased from 1993 kcal/d to 1814 kcal/d, whereas resting fat oxidation increased from 0.04 g/min to 0.06 g/min. His capacity to oxidize fat during exercise increased more than two-fold from 0.24 g/min to 0.59 g/min, while there was a near 3-fold increase in the corresponding exercise intensity that elicited the maximal rate of fat oxidation; 21% *V̇*O_2max_ to 60% *V̇*O_2max_. Hamstring concentric peak torque decreased (1.7 to 1.5 Nm/kg), whereas hamstring eccentric (2.0 Nm/kg to 2.9 Nm/kg), quadriceps concentric (3.4 Nm/kg to 3.7 Nm/kg) and quadriceps eccentric (4.9 Nm/kg to 5.7 Nm/kg) peak torque all increased. Psychological mood-state (BRUMS scale) was not negatively influenced by the intervention and all values relating to the Athlete’s mood-state remained below average over the course of study. This intervention shows that a structured and scientifically supported nutrition strategy can be implemented to improve parameters relevant to bodybuilding competition and importantly the health of competitors, therefore questioning the conventional practices of bodybuilding preparation.

## Background

During bodybuilding competitions individuals are assessed on their physical or ‘aesthetic’ appearance and are usually required to demonstrate a high degree of muscularity and symmetry, as well as low levels of body fat. Careful attention to nutrition and exercise conditioning is undoubtedly important in facilitating the process of becoming ‘competition ready’. Frequently used methods by those preparing for contest include chronic energy restriction, dehydration (water manipulation), sporadic eating and inappropriate use of diuretics and supplements of anabolic steroids and ‘fat burners’ [1]. These methods pose the risk of adverse health consequences that can be physiological (i.e., decreased bone mineral density [2], metabolic disruption [3], increased cardiovascular strain [4]), hormonal [5,6] and/or psychological (i.e. anger, anxiety, loss of eating control/binge eating, pre-occupation with food, short temper [1], mood disturbance [6]), in nature. Competitors may also suffer a reduction in their muscular function, strength and power during the preparation phase of competition [6,7], as physique-oriented objectives are often placed above exercise performance and health goals.

A case study approach has recently been used to outline effective support strategies for the achievement of body composition and/or performance goals in professional boxing [8], professional jockeying [9], and international-standard women’s football [10]. These examples highlight sports (especially boxing and horse riding) where athletes are required to repeatedly manipulate body composition to compete and perform at the highest level, similar to bodybuilding preparation. Despite insightful studies that have documented the physiological changes and dietary practices [6,11,12] that occur during prolonged bodybuilding contest preparation, there have been no case studies that provide a detailed nutrition and conditioning support strategy for the preparation phase of natural bodybuilding competition. Accordingly, we present a 14-week case study demonstrating how a scientifically designed nutrition and conditioning intervention improves body composition, resting and exercise fat oxidation, and muscular strength in an amateur bodybuilding competitor (referred to hereafter as ‘The Athlete’).

## Case presentation

### The Athlete

The Athlete was a 21-year-old male amateur bodybuilder who was aiming to compete in his first bodybuilding competition, UK Bodybuilding and Fitness Federation (UKBFF), in the Men’s Physique category. He had been undertaking bodybuilding training for two years and had not previously sought any conditioning or dietary advice other than that sourced from the Internet and popular fitness magazines. Furthermore, the Athlete was not on any prescribed medication, was a non-smoker and previously supplemented his diet with whey protein only. In the 3 months prior to the intervention his diet was identical on a daily basis; comprising of four meals and two snacks that were high in carbohydrate and protein and very low in fat (Table 1). In addition to the meals he already consumed, he incorporated one ‘cheat meal’ approximately every two weeks, which consisted of one large take-away pizza and one serving (~200 g) of ice cream. His conditioning regime consisted of six to seven days per week of resistance training, focusing on individual muscle groups in each session (total nine hours per week).

**Table 1:** **Example of foods consumed by the Athlete before the intervention**

**Item/description**	**Amount (g)**
**Meal 1**	
Scrambled egg	150
Oats	40 (dry)
**Meal 2**	
Chicken breast	170
Broccoli	150
White rice	40 (dry)
**Meal 3**	
Whey protein	50
**Meal 4**	
Chicken breast	170
White rice	40 (dry)
Sweet potato	150
**Meal 5**	
Chicken breast	170
White rice	40 (dry)
Sweet potato	150
**Meal 6**	
Whey protein	25
Apple	100
**Totals**	
Energy (kcal)	2128
Carbohydrate (g)	212
Fat (g)	28
Protein (g)	257

The Athlete was fully informed of the study aims and potential risks and discomforts following which he provided written informed consent to participate in the study that received full ethical approval from the University of Worcester Ethics Committee.

### Goals of the intervention

The primary goals of the support provided were to: (a) achieve the best possible aesthetic appearance in preparation for UKBFF; (b) improve resting and exercise fat oxidation; (c) preserve muscular function and strength; (d) maintain a positive mood-state during the 14-week lead in to competition.

### Metabolic assessment

Resting Metabolic Rate Assessment (RMR) was determined on six occasions (Familiarization, Baseline, Week 3, Week 8, Week 10 and Week 13) and a graded Exercise Test was completed on three occasions (Familiarization, Baseline and Week 13) to determine rates of fat oxidation during exercise and cardiorespiratory fitness (*V̇*O_2max_). For all assessments the Athlete reported to the laboratory at 07:00 h following an overnight fast from 10 pm the evening before and having abstained from strenuous physical activity, alcohol and caffeine consumption in the 24 h preceding each visit. The Athlete was asked not to perform any physical activity on the morning of testing, such as brisk walking or cycling to the laboratory, and to consume 500 ml water upon waking to encourage hydration. The Athlete was fitted with a facemask for both the RMR and Exercise Testing (Combitox, Drager, Jaeger, Nussdorf Traunstein, Germany) and breath-by-breath measurements of oxygen consumption (*V̇*O_2_) and carbon dioxide production (*V̇*CO_2_) were measured continuously using an online gas analysis system (Oxycon Pro, Jaeger, Wuerzberg, Germany). The gas analyzer was calibrated immediately prior to testing with a known gas concentration (5% CO_2;_ 16% O_2_; 79% N_2_ [BOC Gases, Surrey, UK]) and a three-liter calibration syringe (Hans Rudolf, USA) was used to calibrate the volume transducer. Environmental conditions during testing were: humidity 51 ± 6%; temperature 20 ± 1°C.

For the RMR Assessment the Athlete was required to lie still on a bed in the supine position for 30 minutes in a dimly lit room. There was no visual or auditory stimulation throughout this period. The Exercise Test was completed ~15 minutes after the RMR assessment. This was based on the protocol described previously by Achten and colleagues [13] with the starting speed and inclination of the treadmill (HP Cosmos, Jaeger, Nussdorf Traunstein, Germany) set at 3.5 km/h and 1%, respectively. The treadmill speed was increased by 1 km/h every three minutes until the respiratory exchange ratio (RER) reached 1.00. At this point the treadmill gradient was increased by 1% every minute until volitional exhaustion. Heart rate was measured continuously throughout testing using a heart rate monitor (Polar FT-1, Finland) and was recorded during the final 30 seconds of each exercise stage.

#### Calculations

Resting energy expenditure and fat oxidation were calculated during a stable measurement period i.e., a deviation in *V̇*O_2_ of <10% of the average *V̇*O_2_ between minutes 20–30 (mean ± SD recording period was 5 ± 2 minutes) using the equations of Frayn [14] and a protein correction factor of 0.11 mg/kg/min, as used previously [15,16]. During the Exercise Test *V̇*O_2_ and *V̇*CO_2_ were averaged over the last minute of each sub-maximal exercise stage and fat and carbohydrate oxidation were calculated according to the equations of Frayn [14], with the assumption that the urinary nitrogen excretion rate was negligible. *V̇*O_2_ was considered as maximal when two of the following three criteria were met; an RER >1.1, heart rate within 10 beats of predicted maximum (calculated as 220-age [17]), or an increase of <2 ml/kg/min in *V̇*O_2_ with a further increase in workload. *V̇*O_2max_ was calculated as the highest rolling 60 second average *V̇*O_2_. The results of the Exercise Test were used to create a curve of fat oxidation rate against exercise intensity, expressed as% *V̇*O_2max_. The maximal rate of fat oxidation during exercise (MFO) was determined by visual inspection i.e., by judging the peak of the curve and its corresponding rate of fat oxidation. Fat_max_ was defined as the exercise intensity (%*V̇*O_2max_) that corresponded to MFO, as described previously [18].

### Diet and activity recordings

At Baseline, the Athlete was provided with two sets of digital weighing scales (Electronic Kitchen Scale SF 400 and Swees Digital Pocket Weighing Scales); blank four-day diet log and physical activity diaries; and detailed instructions to enable the completion of these at week 3, 6, 10 and 12. The Athlete was instructed to record all consumed food and drink items accurately and in as much detail as possible. The activity diary was based on Bouchard et al. [19] and required the Athlete to record level-of-activity every 15 minutes, using a code from a 12-point scale (provided). The diary was completed over a 24-hour period on each of the four days. The scale ranged from ‘sleeping’ to ‘vigorous exercise’ and provided examples of activities at each point to assist the Athlete. To ensure the highest possible level of accuracy in estimations of energy expenditure, he was encouraged to make notes on any sport or exercise performed during the four days sampled. Diet logs were analyzed using Nutritics dietary analysis software (Nutritics v3.06, Ireland).

Daily energy expenditure was estimated using the factorial approach [20]. Here, each of the 12 codes, which had a corresponding metabolic equivalent (MET) value, was assigned a Physical Activity Level (PAL) [21] and a daily PAL was determined by multiplying each of the 12 codes by the total amount of time spent at the activity level. Where he had made notes on a specific sport and exercise activity undertaken during the 4 days, the Compendium of Physical Activities [22] was used to calculate the specific PAL value. Daily energy expenditure was then estimated by multiplying the daily PAL value by the age and sex specific resting metabolic rate (RMR; kcal/d) using height, weight and the World Health Organization (WHO) equation [23]:

$$ \begin{array}{l}\mathrm{Energy}\kern0.5em \mathrm{Expenditure}\left(\mathrm{kcal}/\mathrm{d};\kern0.5em \mathrm{men}<30\kern0.5em \mathrm{years}\right):\mathrm{Daily}\kern0.5em \mathrm{PAL}\kern0.5em \mathrm{value}\ast \mathrm{R}\mathrm{M}\mathrm{R}\left(15.4\ast \mathrm{body}\kern0.5em \mathrm{mass}\left[\mathrm{kg}\right]\right)-\hfill \\ {}\left(27.0\ast \mathrm{height}\left[\mathrm{m}\right]\right)+717\Big)\hfill \end{array} $$

### Anthropometric assessment

Baseline assessments are shown in Table 2. Height (Stadiometer, Seca, UK), mass (Seca, UK) and body composition using skin-folds and girths (measured by an International Standards for Anthropometric Assessment (ISAK) Certified Anthropometrist) were assessed at Baseline and at Weeks 3, 5, 7, 10, 12, 13 and 14 of the intervention at a standardized time of 07:00 h. Body fat percentage, fat-free mass (FFM) and fat mass (FM) were calculated using updated sex and race/ethnicity specific equations [24]. All skinfold measurements followed the ISAK i.e., measurements were taken on the right hand side of the body in duplicate or in triplicate if the total error of measurement of the first and second measurement was >5%, following which a mean value was obtained.

**Table 2:** **Anthropometric and physiological characteristics at baseline**

**Characteristic**	**Value**
Age (y)	21
Height (cm)	178.5
Body mass (kg)	86.0
BMI (kg/m^2^)	27
Body fat (%)	14
Fat mass (kg)	11.7
Fat-free mass (kg)	74.3
Maximal oxygen uptake (ml/kg/min)	49.0
Maximal rate of fat oxidation (g/min)	0.24
Fatmax (%*V̇*O_2max_)	21
Resting metabolic rate (kcal/d)	1993

### Additional assessments

The Brunel Mood Scale (BRUMS) [25] was completed at Baseline and at Week 13. Peak torque of the hamstring (flexors) and quadriceps (extensors) of the dominant leg were determined at Baseline and Week 14 at 08:00 h following 24 hours of rest and following meal 1. This was performed on a separate day from RMR and Exercise Testing. A HUMAC NORM Isokinetic Dynamometer (Computer Sports Medicine, Inc. [CSMI], USA) was set up in the seated position, with the adjustments identical on both testing occasions. The Athlete completed three sets of five repetitions of a concentric flexion and concentric extension at 60°/second, with 60 seconds of rest between sets. After 90 seconds of recovery the Athlete completed three eccentric contraction sets of five repetitions flexion and extension at 60°/second, with 60 seconds recovery between sets. For the concentric and eccentric contractions the initial five repetitions of each set were a warm-up, the second set was a familiarization and the final set was performed to maximal exertion. Peak torque values were recorded using the CSMI computer. Prior to isokinetic testing the weight of the limb was weighed to correct for gravity using HUMAC software (CSMI, USA).

### The intervention

To encourage a reduction in fat mass, we chose to obtain an energy deficit through a combination of decreased energy intake and increased energy expenditure. Adjustments in nutritional intake and the quantity of exercise performed during the intervention were made to accommodate the target energy deficit, which was set according the rate of body composition change over the period of study.

#### Diet

**Figure 1:**
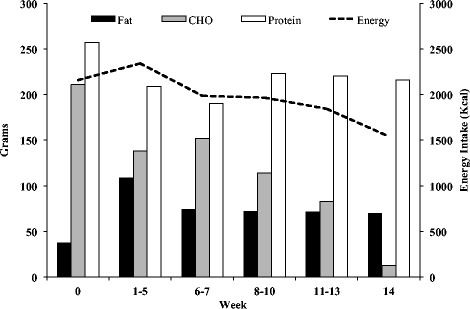
Energy (kcal) and macronutrient (g) intake over the 14-week period of study.

Over the 14-week period of study the Athlete followed a set-meal plan comprising of two menus. Menus 1 and 2 were followed on Conditioning and Rest days, respectively and were designed by the authors who are all certified sports nutritionists (CISSN). Table 3 shows example foods and drinks offered by the two menus and Figure 1 shows energy and macronutrient provision over the 14-week period. A set meal plan was provided for two reasons: 1) it allowed the authors to carefully control energy and macronutrient intake; 2) the Athlete favoured this approach, as opposed to receiving macronutrient and calorie targets, as used in other similar case studies [6,11]. Absolute (relative) carbohydrate, fat, and protein intake over the 14 weeks was 100 ± 56 g/d (20 ± 3% energy), 79 ± 17 g/d (37 ± 4% energy) and 212 ± 13 g/d (45 ± 8% energy), respectively. Carbohydrate recommendations focused on low or medium glycemic index (GI) sources to improve satiety [26] and enhance lipolysis [27]. To enhance muscle glycogen restoration and for purposes of improving meal enjoyment, high GI carbohydrates were also recommended [28]. To improve satiety [26] and help retain FFM and augment fat loss whilst in an energy deficit [29] the Athlete was advised to consume high biological value protein such as chicken and eggs and distribute protein intake throughout the day. This ‘pulsing’ strategy has been found to stimulate daily muscle protein synthesis more effectively than skewing protein intake toward the evening meal [30]. Carbohydrate intake underwent the greatest manipulation over the 14-week period to accommodate the target energy intake (Figure 1). Reducing carbohydrate intake has been suggested as a viable strategy to allow protein intake to remain high in the face of an energy deficit [31]. Fluid suggestions were water, sugar-free cordial and flavored tea that were to be consumed *ad libitum* throughout the day.

**Table 3:** **Menus provided on rest and training days during weeks 1–5**

**Menu 1: Training day**		**Menu 2: Rest day**	
**Item/description**	**Amount (g)**	**Item/description**	**Amount (g)**
**Meal 1**		**Meal 1**	
Venison burger	150	Poached egg	150
Poached egg	150	Oats	50 (dry)
Spinach	50	Whey protein powder	30
**Meal 2**		**Meal 2**	
Whey protein powder	60	Tuna (tinned)	130
Creatine	5	Asparagus	100
Brazil nuts	20	Macadamia nuts	30
**Meal 3**		**Meal 3**	
Mackerel	150	Chicken breast	150
Brown rice	100	Sweet potato	150
Salad leaves	50	Almonds	20
Avocado	50		
Apple cider vinegar	12		
**Meal 4**		**Meal 4**	
Turkey breast	155	Salmon fillet	140
White Basmati rice	100 (dry)	White Basmati rice	50
Mushrooms	100	Broccoli	100
Coconut oil	12	**Snack**	
**Snack**		Chocolate flavored mousse	50
Full-fat cottage cheese	225	Coconut Oil	12
**Totals**		**Totals**	
Energy (kcal/d)	2413	Energy (kcal/d)	2246
Carbohydrate (g)	137	Carbohydrate (g)	143
Fat (g)	119	Fat (g)	96
Protein (g)	207	Protein (g)	212

#### Conditioning

The 14-week conditioning programme is presented in Table 4. Briefly; the Athlete completed four resistance-training sessions (RT) during each week of the intervention; targeting each major muscle group on two occasions per week. Each RT consisted of 6–8 exercises performed for 8–10 repetitions and 4–5 sets [32]. A combination of high intensity interval training (HIIT) and low-intensity steady-state (LISS) exercise, that was performed in the overnight fasted state (07.00-08.00 h), was completed with the aim being to up-regulate oxidative enzyme adaptations to enhance fat utilization [33] and help preserve FFM whilst on a carbohydrate restricted diet [34]. Whilst two recent studies [35,36] show that fasted-state training, compared with fed-state training, does not result in greater losses in fat mass when daily caloric deficit is similar, we prescribed fasted-state training based on the Athletes preference i.e. (i) he found it difficult to perform HIIT and LISS training sessions having eaten in close-proximity (time-wise) to training and (ii) he wanted to consume his morning calories after training, as this gave him something to look forward to. The authors believe these are important practical considerations when prescribing fasted- or fed-state training to athletes.

**Table 4:** **Training program undertaken throughout the intervention period**

**Day (time)**	**Weeks 1-7**	**Weeks 8-10**	**Weeks 11-14**
**Monday (AM)**	Rest	Sprints	Sprints
		10 × 10–15 sec	10 × 10–15 sec
**Monday (PM)**	RT	RT	RT
	Chest and back	Chest and back	Chest and back
**Tuesday (AM)**	Rest	Rest	Incline walk on treadmill
			40 minutes
**Tuesday (PM)**	RT	RT	RT
	Legs	Legs	Legs
**Wednesday (AM)**	Rest	Incline treadmill walk	Incline treadmill walk
		40 minutes	40 minutes
**Wednesday (PM)**	Rest	Rest	RT
			Shoulders and Arms
**Thursday (AM)**	Rest	Rest	Incline treadmill walk
			40 minutes
**Thursday (PM)**	RT	RT	RT
	Shoulders and arms	Shoulders and arms	Shoulders and arms
**Friday (AM)**	Rest	Incline treadmill walk	Incline treadmill walk
		40 minutes	40 minutes
**Friday (PM)**	Circuit training	RT	RT
	30 minutes	Legs	Legs
**Saturday (AM)**	Rest	Rest	Incline treadmill walk
			40 minutes
**Saturday (PM)**	Rest	Rest	Rest
**Sunday (AM)**	Rest	Rest	Rest
**Sunday (PM)**	Rest	Rest	Rest

RT = Resistance Training (the mean duration of each session was 30 minutes).

The number of HIIT and LISS training sessions performed each week was adjusted according to the target energy deficit. In the six weeks prior to competition, “posing practice” was implemented (2–4 times per week), which involved holding isometric contraction of the major muscle groups for 30–60 seconds.

#### Provision of supplements

Whey protein (Optimum Nutrition, Glanbia Plc, Ireland) and one serving of a high protein (with a high whey and casein content), low carbohydrate snack in the late evening (Muscle Mousse, Genetic Supplements, Co. Durham, UK) was provided. Creatine monohydrate (Optimum Nutrition, Glanbia Plc, Ireland) was given as 20 g per day for the first five days of the intervention, followed by 5 g per day for 93 days [37].

### Evaluation of the intervention and commentary

**Figure 2:**
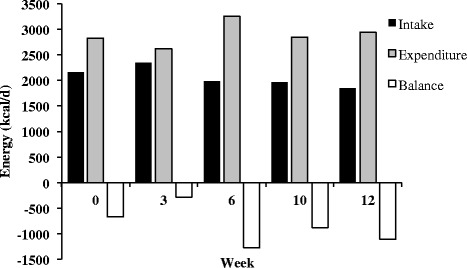
Recorded energy intake and predicted energy expenditure and energy balance.

**Figure 3:**
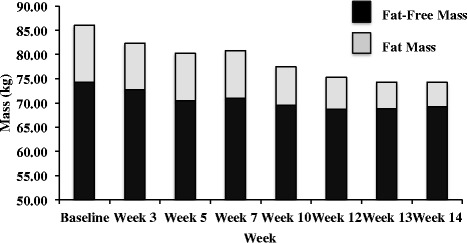
Anthropometrical changes over the 14-week period of study.

**Figure 4:**
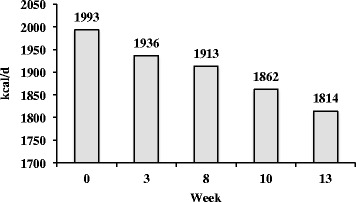
Resting metabolic rate as a function of time.

Recorded energy intake, predicted energy expenditure and energy balance (Figure 2), and anthropometric changes (Figure 3) over the 14-week period are shown. There was a reduction in RMR over the course of the preparation period, which is consistent with previous reports that have shown a decline in RMR during periods of caloric restriction [6,38,39] (Figure 4).

Over the course of the 14-weeks total body mass loss was 11.7 kg’s. This equated to an average weight loss of 0.98%/week, which is in accordance with the recommended weekly rate of 0.5-1.0% [40]. The energy deficit was 882 ± 433 kcal/d and this led to a reduction of 6.7 kg’s (or 6.8% body fat or 33 mm using sum of 8 skinfolds) and 5.0 kg’s, in fat mass and FFM, respectively. It is not uncommon for individuals to lose FFM whilst in a negative energy balance [8,9]. Resistance training, HIIT, creatine monohydrate supplementation, and high-protein diets have all been reported to promote FFM accretion and prevent FFM loss during energy restriction (for review, see Churchward-Venne, Murphy, Longland, & Phillips [41]), however these strategies were not sufficient to prevent the decline observed in our study.

One potential reason why the Athlete lost FFM in spite of the abovementioned approaches could be because of the size of the energy deficit was larger than that applied by others. For example, one case study observed an increase (albeit only slight, 0.45 kg) in FFM during a period of less severe caloric restriction (mean ± SD energy deficit: −343 ± 156 kcal/d) [10]. A weight loss strategy that is more gradual might have induced a lesser reduction in FFM. For instance, the athlete studied in Kistler et al. [11] dieted at a rate of ~0.7% bodyweight per week and had a higher percentage of FFM loss (32% of total body mass loss) than Rossow et al. (21% of total body mass loss) [6] where the athlete dieted at a rate of ~0.5% bodyweight per week. The Athlete in this study dieted at a rate of ~1.0% bodyweight per week and had the highest percentage of FFM loss (43% of total body mass loss). This suggests that the rate of weight loss might influence the percentage of FFM loss, even within the 0.5-1.0% bodyweight per week range as previously recommended [40]. Indeed, there is greater potential for loss of FFM as adipose tissue declines. Accordingly, an additional strategy to offset reductions in FFM whilst in energy deficit might be to reduce the size of the energy deficit as competition nears [40]. This strategy would require that the athlete allows sufficient time to reach a desirable level of body fat, such that when competition nears they do not need to place themselves in a large energy deficit to reduce body fat. Furthermore, the Athlete consumed (mean ± SD) 37 ± 4% energy from fat and 20 ± 3% energy from carbohydrate over the duration of the intervention. This is less carbohydrate and more fat (expressed as% energy) than was consumed by the athletes in Rossow et al. [6] and Kistler et al. [11] and has previously been recommended [40]. It has been shown that loss of FFM is minimal when ample carbohydrate is consumed and dietary fat reduced during a period of caloric restriction [42,43], which may offer one further reason why the Athlete lost more FFM than in other similar studies [6,11]. Finally, the Athlete undertook more endurance-based exercise than in Rossow et al. [6]. Previous work suggests that the amount of endurance exercise performed is associated with interference in muscle strength and size gains [44] and this could also provide a reason why the Athlete in our study lost a higher percentage of FFM than in Rossow et al. [6].

#### Maximal oxygen consumption and substrate metabolism

Absolute and relative *V̇*O_2max_ at Baseline was 4.2 L/min and 49 ml/kg/min, respectively. At Week 13, this decreased to 3.4 L/min and 46 ml/kg/min, respectively. Resting heart rate reduced from 54 beats per minute (bpm) at Baseline to 37 bpm at Week 13. Rossow and colleagues [6] and Kistler et al. [11] observed similar reductions in absolute *V̇*O_2max_ and resting heart rate during a period of competition preparation in a professional male bodybuilder. In contrast to our findings, these studies [6,11] reported an increase in relative *V̇*O_2max_ (42 ml/kg/min to 45 ml/kg/min and 42 ml/kg/min to 48 ml/kg/min, respectively).

Resting RER declined from 0.87 at Baseline to 0.82 at Week 13. Accordingly, resting fat oxidation increased from 0.04 g/min (0.53 mg/kg FFM/min) to 0.06 g/min (0.83 mg/kg FFM/min) from Baseline to Week 13, respectively, which translated to an increase in the relative contribution of fat to energy expenditure at rest by 18% from Baseline (33% of energy) to Week 13 (51% of energy). The capacity to oxidize fat during the Exercise Test also improved, as the MFO arising from the GET increased from 0.24 g/min at Baseline (3.23 mg/kg FFM/min) to 0.59 g/min (8.58 mg/kg FFM/min) at Week 13, demonstrating a greater than two-fold increase in the Athlete’s capacity to oxidize fat during exercise come the end of the intervention. The increased capacity for fat oxidation during the Exercise Test was also apparent in other ways: The number of submaximal stages completed with a RER < 1.0 increased two-fold from Baseline (5 stages) to Week 13 (10 stages). Further, Fatmax increased 39% from Baseline (21% *V̇*O_2max_) to Week 13 (60% *V̇*O_2max_). Improvements in fat oxidation could help protect towards developing an unfavourable cardiometabolic phenotype. For example, cross-sectional studies show that a reduced capacity to oxidize fat during exercise is associated with a higher clustering of metabolic syndrome risk factors [45] and unfavourable fat mass distribution (higher abdominal to lower body fat mass index, associated with metabolic disorders such as insulin resistance and dyslipidaemia [46]). Moreover, impairments in fat oxidation at the level of skeletal muscle have been associated with reduced metabolic flexibility and insulin resistance [47]. Whilst one might argue that this has little bearing on the subjective outcome of a bodybuilding competition, it is undoubtedly important for athletes and practitioners to consider the health effects of dietary and conditioning approaches. From the perspective of body composition, previous work links a high daily respiratory quotient (RQ), indicative of a low relative fat oxidation, with an increased risk of body mass gain [48] and body fat mass regain after diet-induced weight loss [49]; independent of energy expenditure. Recent research has shown that those who exhibit a higher capacity to oxidise fat during exercise (i.e. a higher MFO) also demonstrate a higher 24-hour fat oxidation and greater insulin sensitivity [50], and therefore it is not unreasonable to consider a high capacity to oxidise fat whilst physically active as beneficial for the long-term maintenance of body composition and metabolic health. Furthermore, there is some evidence that a lower use of fat as fuel during exercise [51] and on a 24-hour basis [52,53] is predictive of increased *ad libitum* energy intake, which may offer a behavioral explanation linking low fat oxidation to unfavorable body or fat mass development.

#### Strength and power

At Baseline, hamstring concentric and eccentric peak torque was 146 Nm (1.7 Nm/kg) and 172 Nm (2.0 Nm/kg), respectively. At Week 14, hamstring concentric strength decreased to 114 Nm (1.5 Nm/kg), whereas hamstring eccentric strength increased to 218 Nm (2.9 Nm/kg). At baseline, quadriceps concentric and eccentric peak torque was 293 Nm (3.4 Nm/kg) and 424 Nm (4.9 Nm/kg), respectively. At Week 14, absolute quadriceps concentric strength declined to (273 Nm) but increased when expressed relative to body mass (3.7 Nm/kg). Absolute quadriceps eccentric peak torque remained similar (423 Nm) but increased when considered relative to body mass (5.7 Nm/kg). Taken collectively, these findings show that the alterations in body composition observed in the present study i.e., reduced fat mass and FFM, did not compromise most measures of strength. An allied observation was made by Rossow et al. [6] who demonstrated that relative measures of strength remained similar over a six-month preparation phase, however absolute measures of strength declined (note that their method of strength testing was bench, deadlift and squat performance), which is similar to Bamman et al. [7] who observed a significant reduction (−129 N) in isometric deadlift force following a 12-week preparation period. Whilst it could be argued that strength is not essential to bodybuilding competition performance, the ability to maintain an appropriate level of strength is important for the achievement of physique-oriented goals. Indeed, Rossow et al. [6] reported that the decrements in strength observed in their study required four to six months to return to baseline and suggested that it is preferable to maintain strength during the preparation phase of competition to facilitate a quick return of training competency post-competition.

#### Subjective ratings of health and performance

**Figure 5:**
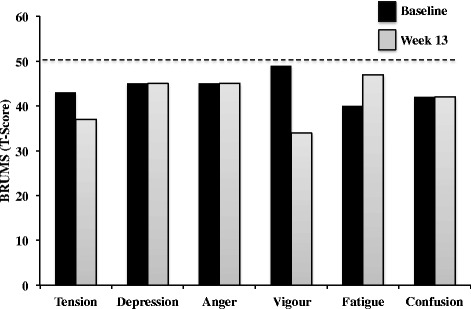
BRUMS scale pre- and post-intervention.

Over the 14-week period of study, the Athlete reported no severe feelings of hunger or thirst. Previous work suggests that rigid dietary regimes, as opposed to ones that are more flexible, are associated with a higher prevalence of overeating and binging [54,55], however the Athlete in this study reported no such desires. It could be that there are large inter-individual differences in the response to particular dietary and training regimes, which might offer one explanation for the apparent discrepancy. Compared with Baseline, the Athlete described more stable energy levels, concentration and focus throughout the day as well as during conditioning sessions. These positive outcomes were reflected in the BRUMS assessment, which showed anger, confusion, depression, fatigue and tension all remained below average at the end of the intervention, whilst fatigue increased slightly from Baseline to Week 13 but remained below average (Figure 5). These favorable findings are in contrast to Rossow and colleagues [6] who reported that total mood disturbance increased in the six months leading up to competition and did not return to baseline until four months after competition. Compared with Rossow et al. [6], the Athlete in our study followed a strict meal plan, which induced a faster rate of weight loss and required a higher volume of cardiovascular training, all of which would suggest that contest preparation in the present study may also have had detrimental effects on mood, however no such decrements were reported.

## Conclusions

There is a wide range of speculation regarding optimal nutrition and conditioning strategies to facilitate the achievement of an optimal figure for physique-orientated competitions such as bodybuilding. Furthermore, it has previously been suggested that some of the unfavourable physiological alterations that accompany the achievement of an optimal physique during the preparation phase i.e., a reduction in mood-state, muscular strength and power and physical performance, are a prerequisite [6]. This case study shows that a structured and scientifically supported nutrition and conditioning strategy can be effectively implemented to reduce body fat whilst improving physiological parameters of health, maintaining a favourable mood state and positively influencing strength. Here, the Athlete consumed four meals and one snack on each day of preparation. The majority of the Athlete’s nutrition came from whole foods and there was minimal reliance on dietary supplements, with the exception of those that have only strong scientific evidence in support of their ergogenic effects. Taken collectively, and in contrast to popular myth, our case study shows that it is not necessary to skip meals, neglect specific macronutrient groups, dehydrate or consume a large variety of supplements to adequately prepare for bodybuilding competition. Nevertheless, we acknowledge that 43% of the total body mass lost was FFM, which is not a favourable response. Accordingly, we propose a variety of practical strategies to assist in counteracting this. Whilst the authors appreciate that a limitation of the present study is its sample size of 1, this approach enabled us to accurately document a variety of measures that may not have been possible to acquire using a randomized control trial in a laboratory. As such, we offer this case study as a real-world applied example for other male bodybuilding competitors and coaches seeking to deploy nutrition and training strategies. Finally, the Athlete placed 7^th^ out of 19 competitors, which he and the support team acknowledged as a successful performance given that this was his first time competing as a physique competitor.

## Consent

Written informed consent was obtained from the patient for publication of this Case report and any accompanying images. A copy of the written consent is available for review by the Editor-in-Chief of this journal.
